# Ameliorative role of silver nanoparticles incorporated with chitosan solution and leukocyte platelet-rich fibrin scaffold during colon anastomosis in rabbits

**DOI:** 10.1007/s10856-025-06908-0

**Published:** 2025-07-08

**Authors:** Mohammed Albahrawy, M. I. EL-Henawey, Ayman S. Elmezayyen, Esam Mosbah, Gamal Karrouf, Adel Zaghloul, Marwa Abass

**Affiliations:** 1 Surgery, Anesthesiology and Radiology Department, Faculty of Veterinary Medicine, Mansoura University, Mansoura, Egypt; 2 Physics Department, Faculty of Science, Mansoura University, Mansoura, Egypt; 3 Physics Department, Faculty of Science, New Mansoura University, New Mansoura, Egypt

## Abstract

This research aimed to detect the experimental value of silver nanoparticles (AgNPs) loaded with chitosan (CS) and leukocyte platelet-rich fibrin (L-PRF) in promoting the healing process of the anastomotic colon in rabbits. Forty-two healthy male white New Zealand rabbits underwent total transection and anastomosis of the ascending colon. The rabbits were then randomly divided and equally allocated into two groups (*n* = 21): the control (C) group, in which the transected colon was traditionally anastomosed with simple interrupted sutures, and the treated (T) group, in which the colon was traditionally anastomosed and completely wrapped by a L-PRF scaffold that was inoculated with 1 ml of AgNPs-CS solution. Clinical macroscopic observations of rabbits and pain scale scores were evaluated postoperatively on the 7th,14th, and 28th days. Additionally, seven rabbits from each group were euthanized at each time point to assess bursting pressure, radiographic stenotic degree, histopathological findings, and immunohistochemical examination. The anastomotic colon in the T-group revealed a significant improvement in the overall health status of rabbits, as well as mechanical bursting pressure scores. Pain scores and radiographic stenotic degree were significantly lower in the T-group. Moreover, the histopathological examination of the anastomotic colon in the T-group showed an increased collagen deposition in the submucosa, proper re-epithelialization of the mucosa, higher angiogenesis, reduced adhesions, and significantly higher histopathological healing scores than the C-group. The AgNPs-CS/L-PRF scaffold successfully reduced complications following colon anastomosis and potentially ameliorated the healing process of the anastomotic colon in the experimental rabbits.

**Graphical Abstract:**

**Figure:**
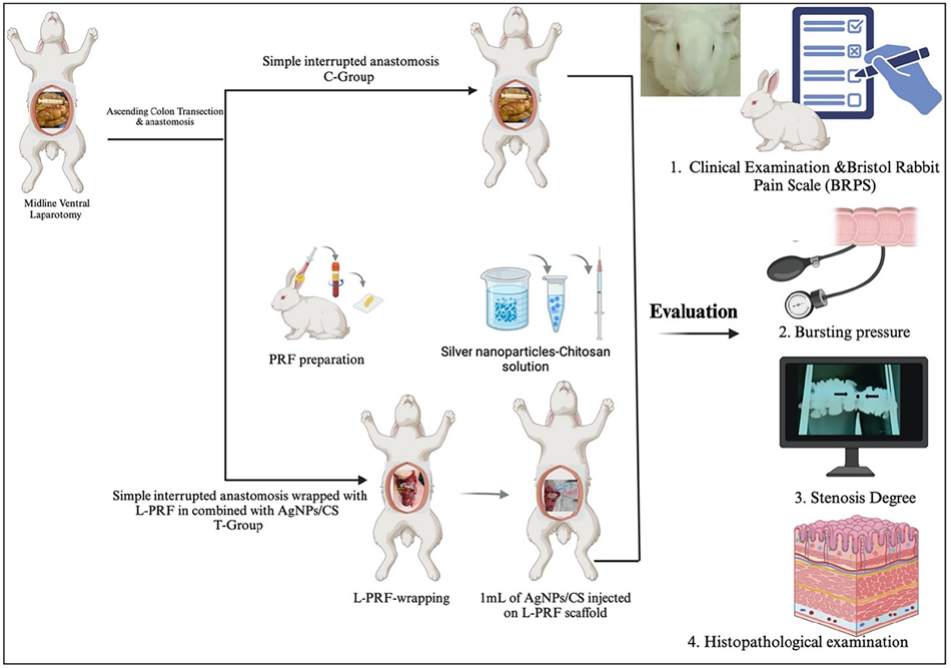
Schematic cartoon of the experimental study. The authors of the manuscript designed it. (Created with Biorender.com with permission).

## Introduction

Intestinal anastomosis is one of the most often performed surgical operations in Gastrointestinal surgeries [1]. The primary concerns were, and still are, discomfort, increased mortality, longer hospital stays, and life-threatening consequences from poor wound healing in the intestinal anastomoses [2]. Among surgical operations within the gastrointestinal tract, the colon is considered more challenging than those in the small intestine or stomach [3]. Studies have shown that colon anastomoses have a leaking rate of up to 30% [4].

A potentially dangerous clinical complication that can occur after colorectal anastomosis (3–20%) is anastomotic insufficiency and leakage, which raises the risk of postoperative morbidity and mortality [5–7]. Previous research has focused on using natural biological regeneration substances to enhance the healing process, such as platelet capabilities [8], bioactive molecules like chitosan (CS) [9], and nanomaterials like silver nanoparticles (AgNPs) [10].

Platelets are not only essential for hemostasis, but they also play a role in the process of wound healing [11]. They have several healing sources, including inflammatory and healing cytokines, as well as growth factors [12]. Therefore, it is believed that applying concentrated platelets could hasten the healing process. Various preparations high in platelets have been tested to accelerate tissue repair, yielding promising outcomes. Leukocytes are in leukocyte platelet-rich fibrin (L-L-PRF), a hard fibrin biomaterial. The L-PRF matrix contains almost all the platelets and over 50% of the leukocytes from the initial blood sample. It provides a robust fibrin construction and a particular three-dimensional spreading of the platelets and leukocytes [12]. In the early stages of this process, the interaction between these cells and the fibrin matrix is encouraged by the gradual release of growth factors for up to fifteen days, leading to accelerated wound healing [13].

Chitosan (CS), the principal derivative of chitin, is a copolymer of glucosamine and N-acetylglucosamine units linked by glycosidic bonds [14]. It has a hemostatic effect [15] and is considered a biocompatible, biodegradable, non-toxic, biologically adhesive, and antibacterial material [16]. Nanotechnology-driven therapeutic interventions displayed the prospective features of assisting wound healing and eventually repairing the injured tissue [17]. AgNPs are the best-known nano products [18] and have attracted considerable attention as antimicrobial agents with proper long-term anti-inflammatory properties, especially in a peritoneal adhesion model [19].

This research aimed to find an effective method to prevent leakages after anastomoses, reduce adhesions, and provide sufficient mechanical strength around the anastomotic wound. Additionally, it aimed to investigate the potential bio-stimulatory synergistic effects between AgNPs and CS loaded in the L-PRF scaffold after wrapping around the anastomotic colon in rabbits. This experimental study hypothesized that the AgNPs-CS loaded in the L-PRF scaffold could reinforce the healing of the anastomotic colon and reduce postoperative complications in rabbits.

## Materials and methods

### Calculation of the sample size

The G-Power 3.1.9.4 software was utilized to calculate the accurate sample size needed to test the research hypothesis. The results showed that to get 95% power for finding a medium effect size, with a critical F-value of 4.0847, a significance level of α = 0.05, and an effect size of f = 0.46, the size of the sample was *N* = 40 for ANOVA: repeated measures were required. Therefore, the sample size was *N* = 40, which was sufficient for testing the research hypothesis. This study was approved by the Ethics Committee of Mansoura University Animal Care and Use Committee with the code number MU-ACUC (VM.PhD.22.11.6). All of these research methods were under the ARRIVE guidelines.

### Animals

Forty-two healthy male white New Zealand rabbits were purchased from the Faculty of Agriculture, Mansoura University. The age of the rabbits was 5 ± 1.6 months, and their weights were 2.5 ± 0.5 kg. All rabbits were accommodated separately in the experimental animal chamber at 55% humidity, a persistent temperature of 22° ± 1 °C, and a 12-h light/dark cycle. All animals had free access to ordinary laboratory nutrition and water as required, and they were adapted for fourteen days before beginning the experiment. This study was done at the experimental laboratory animal chamber of the Surgical Department of Mansoura Veterinary Teaching Hospital, Faculty of Veterinary Medicine, Mansoura University. The animals were randomly equally allocated into two groups (*n* = 21): the control group (C-group) and the AgNPs-CS/L-PRF treated group (T-group).

### Leukocyte platelet-rich fibrin (L-PRF) preparation

L-PRF was prepared as described by [12]. Eight milliliters of autologous blood were initially collected from each rabbit using vacutainer needles before the experimental surgery. The blood sample was then divided into two 4-milliliter plain blood tubes. These tubes were centrifuged at 2700 rpm for 12 min. Following the centrifugation, the tubes were held vertically for 5 min. Each tube consisted of three layers: white-yellowish acellular plasma at the top, clotted blood at the bottom, and the fibrin mass in between. Using sterile thumb tissue forceps, the L-PRF was carefully picked up, separated from the clotted blood, and placed on sterile gauze.

#### Silver nanoparticles and chitosan solution (AgNPs-CS) preparation

A silver nanoparticle-chitosan solution was prepared using electrochemical techniques. Chitosan powder (1% w/v) was dissolved in a 1 M aqueous citric acid solution (7% w/v) to create a chitosan solution. The potentiostatic method was utilized at a constant potential of 1.5 V to synthesize nanoparticles electrochemically. The used cell consists of two electrodes: one sliver sheet (anode) and the other platinum sheet (cathode), positioned 1 cm apart and immersed vertically in the electrolytic solution. The process was carried out at ambient conditions with magnetic stirring. The reaction mixture was constantly swirled until all of the silver ions had been reduced to AgNPs. This was indicated by a shift in the solution’s color from translucent to yellow. The entire procedure was carried out in complete darkness at a constant stirring rate of 450 rpm [20].

#### Transmission electron microscope (TEM) analysis

The size and morphology of AgNPs were investigated using a TEM connected to a charge-coupled device (CCD) camera. The samples were sonicated using an ultrasonic cleaner and sonicator for evaluation. To prepare the samples for TEM imaging, a drop of the solution was placed onto a carbon-coated copper grid. The solvent was allowed to slowly evaporate at room temperature before capturing the TEM images.

**Fig. 1:**
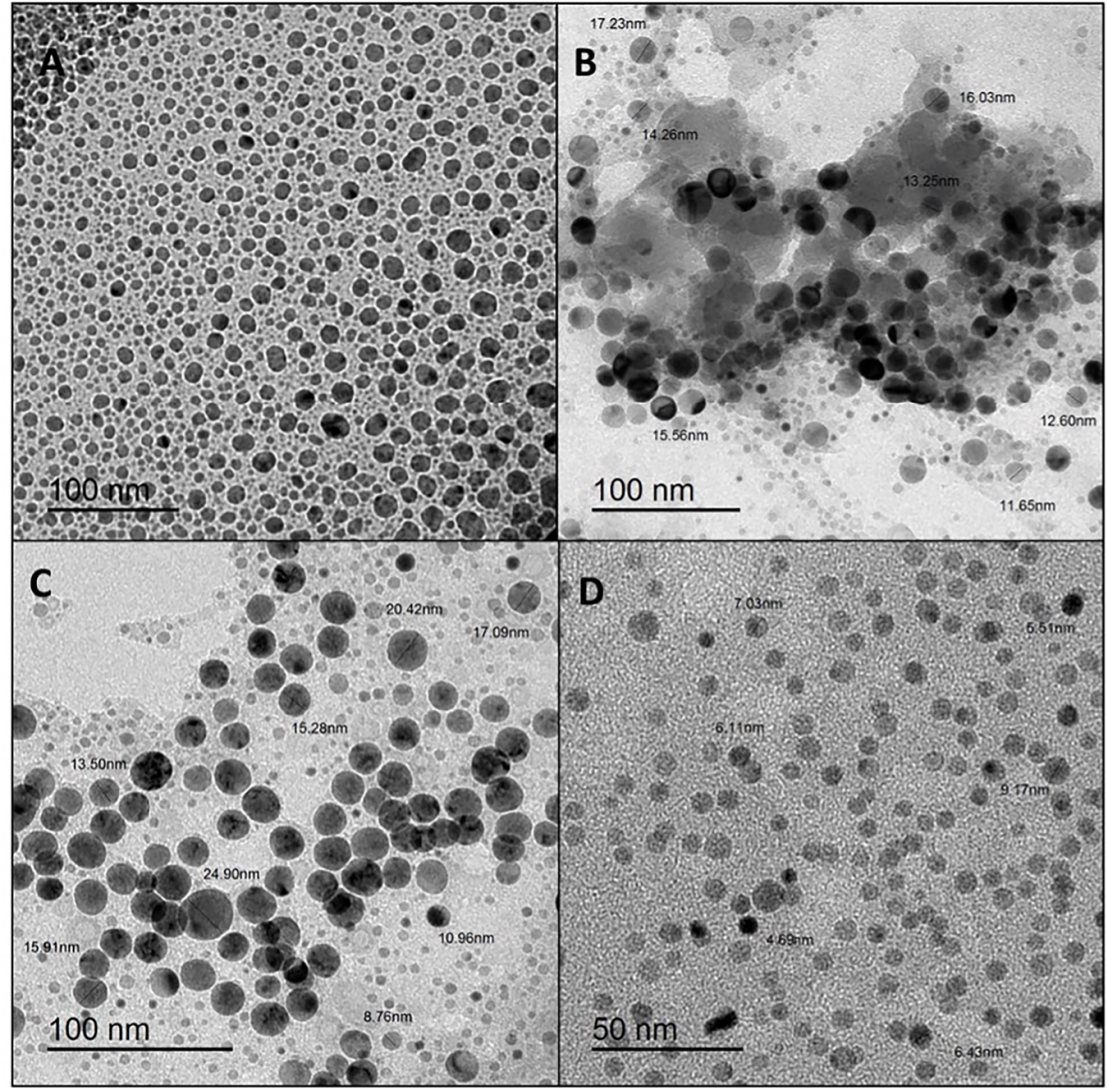
Transmission electron microscopy (TEM) images of different sizes of AgNPs-CS at various magnifications (**A**–**C**) 100 nm, and **D** 50 nm

Scanning Electron Microscopy analysis (SEM) shows the formation of spherical silver nanoparticles within the chitosan matrix after two hours of electrosynthesis. On the other hand, the estimation of the size distribution of the AgNPs revealed that they were very well-distributed, with an average size of 4.2 nm. The small size of the silver nanoparticles can be attributed to the high capping agent ability of chitosan, which effectively surrounds the nano-silver particles, preventing them from aggregating [20] (Fig. 1A–D).

**Fig. 2:**
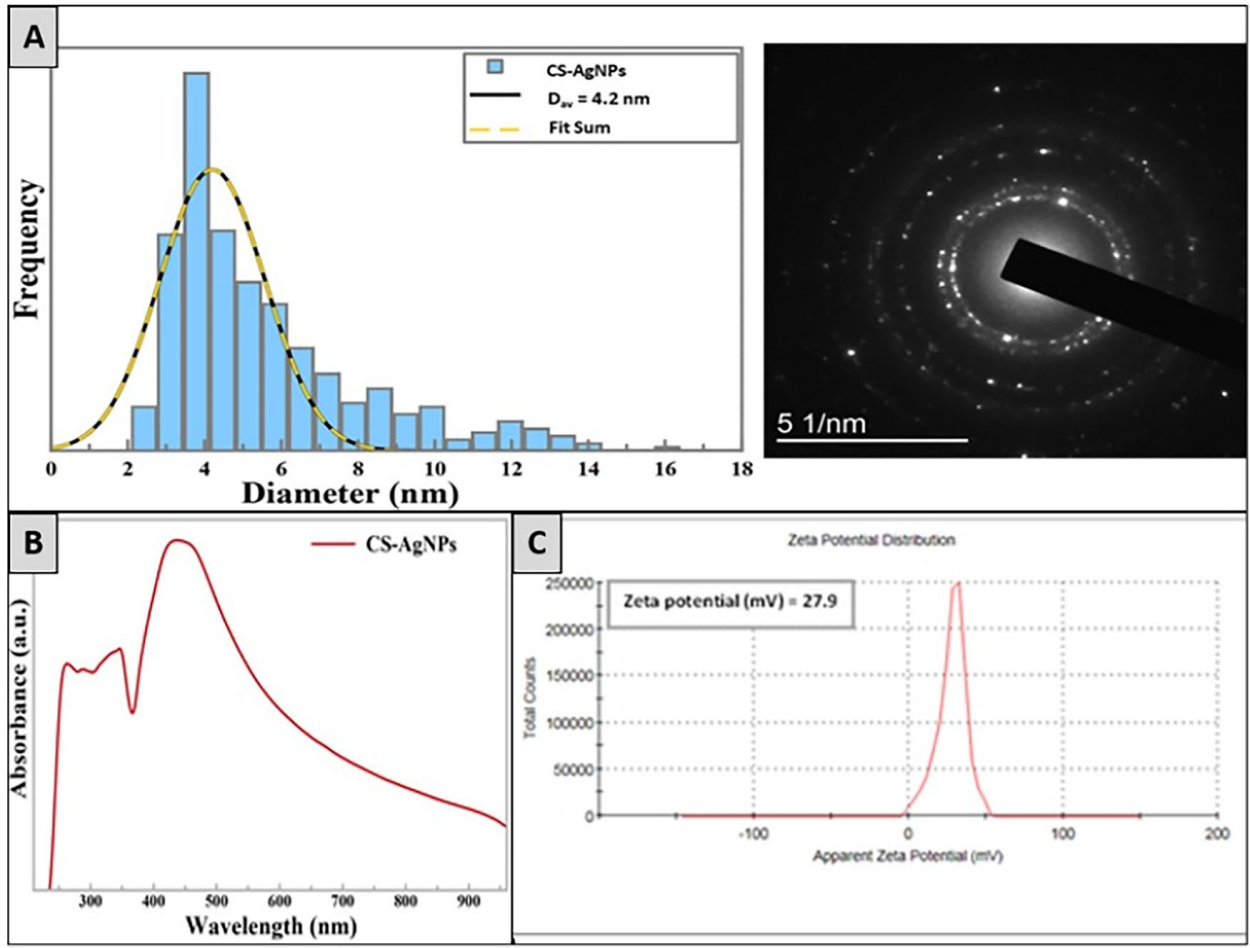
**A** A representative SAED pattern of AgNPs-CS. **B** UV-visible spectroscopic measurement of AgNPs-CS. **C** Zeta potential measurements for AgNPs in polymer matrix CS

The observed ring patterns, combined with many diffraction spots, suggest that the produced AgNPs have a polycrystalline structure. This is a characteristic of a selected area electron diffraction (SAED) pattern, as shown in the inset (Fig. 2A).

#### Ultraviolet (UV)—visible spectroscopy

UV visible spectroscopy established the foundation for the synthesis of AgNPs. A double-beam spectrophotometer was used to detect UV-visible light with a wavelength accuracy of ±0.3 nm within the range of 190–1100 nm. A 1-centimeter quartz cuvette holder was used in the measurements for room temperature samples. To observe the AgNPs SPR peaks, 4 mL of the solution was placed in a quartz cuvette.

The electrochemical production of silver nanoparticles in situ inside the chitosan solution’s electrolyte has been examined using UV-vis spectroscopy. The translucent tint of the electrolyte solution changed to a pale yellow during the electrochemical process and eventually turned yellow as the electro-synthesis time increased [20]. This color change could be a precursor to the formation of silver nanoparticles, as supported by UV-Vis studies. UV-visible spectroscopy is commonly used as an analytical method to study the creation of silver nanoparticles during synthesis due to the distinctive surface plasmon resonance observation.

The surface plasmon resonance of AgNPs was responsible for the occurrence of a single maximum peaking at 420 nm [21], indicating the presence of spherical nanoparticles. This was also confirmed later by TEM results. Additionally, an absorption peak that occurred in parallel at 250 nm may be attributed to the synthesis of [chitosan/pullulan]-complex Ag^+^ [22] (Fig. 2B).

#### Zeta potential analysis

The Zeta size Nano-ZS90 was used to assess the surface charge of a diluted solution made with deionized water. The surface charge distribution was obtained at room temperature by averaging data collected in triplicate.

When assessing the stability of nanoparticle suspensions, the zeta potential is an essential characteristic to consider. Furthermore, it provides important details regarding the location of charged groups on the polymer chains surrounding the nanoparticles. After two hours of electrochemical processing, the measured zeta potential for silver nanoparticles in the polymer matrix CS was 27.9 ± 9.35 mV [23]. The increased availability of the protonated NH3+ groups directed toward the solution gives the silver nanoparticles their positive surface charge. As a result, there will be strong electrostatic repulsion between the particles, improving their stability [24] (Fig. 2C).

#### Inductively coupled plasma analysis (ICP)

The technique known as inductively ICP is a valuable and well-acknowledged method for identifying and documenting individual components present in a sample. ICP was used to determine the concentration of Ag. With a concentration of 1196 mg/L, it is believed that the silver in AgNPs-CS is appropriately electro-released within the polymeric matrix. So, the silver concentration in 1 ml of chitosan solution was 0.12%.

### Colon transection and anastomosis

All rabbits were injected intramuscularly (IM) with Cefazolin (22 mg/kg, 1 gm, Hikma Pharmaceuticals, Egypt) and intravenously (IV) with metronidazole (10 mg/kg, 5%, FLAGYL, Pioneer Pharma, Egypt). Additionally, a 24-gauge intravenous polyurethane catheter (Cat. 1001, ULTRA, Egypt) was inserted into the peripheral ear vein 60 minutes before the surgery. The rabbits were then anesthetized by IM injections of ketamine hydrochloride (45 mg/kg, 10%, Ketalar, Pfizer, US), Midazolam (2 mg/kg, 5%, Midazolam, Pfizer, US), and xylazine (2 mg/kg, 2%, XYLAJECT, ADWIA CO., Egypt).

The rabbits were placed in the dorsal recumbency, and the ventral abdominal region was aseptically prepared by shaving the fur and disinfecting it with 70% alcohol and 10% povidone-iodine. An approximately 5 cm-long ventral midline celiotomy incision was then made, followed by a thorough examination of the abdominal cavity to rule out any abnormalities. The ascending colon was identified, exteriorized, and then toweled with a sterile drape soaked in warm saline at 37 °C to prevent heat loss and dryness. Ten cm away from the end of the cecum, the colon was surgically transected, and then the two terminal ends were carefully cleaned of ingesta using moistened aseptic swabs before being sutured. In the C-group, the terminal segment of the transected colon was anastomosed end-to-end using a single layer of simple interrupted stitches with 5/0 polyglyconate (Maxon™ Monofilament Absorbable Suture, UK).

**Fig. 3:**
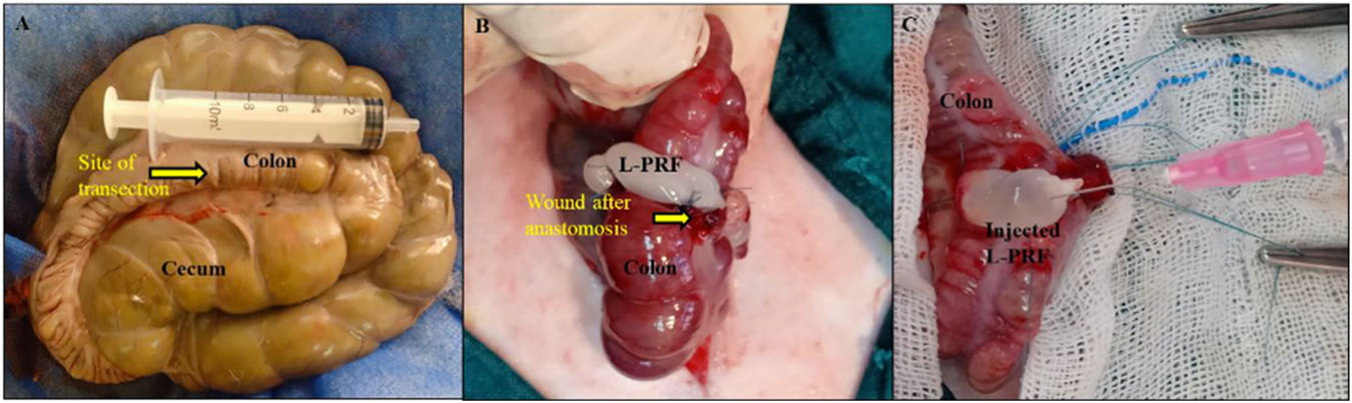
**A** Site of ascending colon transection. **B** L-PRF clot fixation on anastomotic colon site (yellow arrow). **C** L-PRF after CS-AgNPs injection

In the T-group, the L-PRF scaffold completely encircled the anastomotic line of the colon after suturing. The two free ends of the L-PRF were sutured with a 5/0 Maxon suture, and an additional stitch was applied in the middle of the L-PRF scaffold to prevent sliding. Subsequently, 1 mL of AgNPs-CS solution was carefully and evenly inoculated into the L-PRF scaffold using an insulin syringe (Fig. 3).

Before suturing the layers of the abdominal wall, the abdominal cavity was washed with normal saline. The anastomotic line was checked for leakage and luminal continuity. The muscles of the abdomen were sutured using continuous stitches with 3/0 polyglyconate. Finally, 2/0 polypropylene sutures (Demotech, USA) were used in an interrupted suture pattern to suture the skin.

Postoperatively, every rabbit was placed in its kennel. Following the surgery, food was withheld for 24 h before gradually reintroducing it over the next three days. Povidone iodine was applied to the wounds daily. Prophylactic antibiotic treatment of 22 mg/kg of cefazolin was injected for five days. Furthermore, meloxicam as an anti-inflammatory (0.5 mg/kg, 2%, METACAM, Pipeline Pharma, Egypt) was administered IV for three successive days.

### Wound healing assessment

All of the following evaluation parameters were performed blindly by an experienced veterinarian.

#### Post-operative clinical evaluation

A clinical and physical examination of the rabbits was conducted daily from day 0 to day 28 post-operation. This included observing urination, defecation, the general status of the rabbits, and wound inspection.

According to the Bristol Rabbit Pain Scale (BRPS) [25], pain scores were assessed before the operation (day 0) and on the 1^st^, 7^th^, 14^th^, and 28^th^ days postoperatively. The BRPS is a composite pain score that includes six items (grooming, posture, locomotion, demeanor, ears, and eyes) with four levels of pain degree (zero, one, two, and three), resulting in a total BRPS score ranging from zero to eighteen.

Seven rabbits from each group were humanly euthanized by rapid IV administration of 150 mg/kg of thiopental sodium (Anapental, 500 mg, Sigma, Egypt) at the 7^th^, 14^th^, and 28^th^ days post-operation.

Subsequently, a laparotomy was performed to identify the colonic anastomosis, make gross observations, and note the presence of any adhesions. Then, the colon containing the anastomosis line was carefully removed for an approximately 8 cm length resection without causing any damage to it.

#### Postmortem findings

The adhesion numbers and scores between the abdominal organs or wall and colon were assessed [26]. The adhesion score ranged from zero to four. “Zero” represents no adhesion, “One” represents one adhesion among the abdominal organs or between the abdominal organs and the abdominal wall, “Two” represents two adhesions between the abdominal organs and the abdominal wall, “Three” represents more than two adhesions between the abdominal organs and the abdominal cavity, and “Four” represents an adhesion conglomerate.

Anastomotic leaks were evaluated using the anastomotic leakage scores [27]^.^ These scores ranged from zero to three: “Zero” indicated the absence of leakage, “One” indicated anastomotic abscesses, “Two” indicated big abscesses or free pus, and “Three” indicated either obvious dehiscence or fecal peritonitis.

#### Anastomotic bursting pressure (BP)

The bursting pressure of each anastomotic segment was measured [28] to determine its strength. An approximately 8 cm colon segment, which included the anastomotic line and any adhesions (if present), was carefully removed without damaging the anastomotic line, and the intraluminal fecal material was evacuated. Two Doyen intestinal forceps were used to clamp the two ends of the colon segment, and a butterfly catheter attached with a three-way stopcock was inserted into the bowel.

The other sides of a three-way stopcock were connected to the infusion pump and the pressure gauge device to determine the bursting pressure. The colon segment was kept submerged, and pressure was raised by continuously injecting 2 ml/min. of water. The BP was determined by taking the maximum pressure (mm Hg) that was recorded just before the abrupt loss of pressure.

#### Stenosis degree (SD)

A contrast X-ray was applied on the colon segment using barium sulfate solution (Barium Sulfate, 25%, Hekmia Company, Egypt) at 50 kilovolts and 3 milliamperes per second. Using the formula [29] to determine the degree of stenosis in the anastomotic segment. ${\rm{Narrowing}}=100\times \left(1-\frac{2{\rm{A}}}{{\rm{B}}+{\rm{C}}}\right)$

**A** is the diameter of the colon in the anastomotic line. **B** and **C** are the diameters of the colon 2 cm after and before the anastomotic line.

#### Histopathological examination

The colon segment was incised vertically, and about 10 mm surrounding the suture line was collected for microscopical evaluation. The anastomotic colon was cut and fixed in 10% neutral buffered formalin from each rabbit. The tissue samples were dehydrated, cleaned, and immersed in paraffin. A 5-mm section was then performed and stained with hematoxylin and eosin (H&E) and Masson trichrome (MT). Using a light microscope to examine the stained slides and evaluate the following scores.

The severity of colon wall destruction, the affected layers, and the extent of damage and lesions in the colon wall were all scored [30]. The lamina propria, submucosa, and tunica muscularis underwent a histomorphological assessment as outlined in [31]. The score for epithelialization development of the colon wall, the affected layers of the colon wall, the extent of damage and lesions, and the score of inflammation at different layers of the colon wall were determined based on guidelines [32]. Using MT-stained slides, the amount of collagen deposition in anastomotic tissue was evaluated and scored as absent, mild, moderate, or confluent cell fibers according to [33]. The mean histology score for each group was calculated by adding together all of the scores (Table 1).

**Table 1:** The histopathological assessment scores of the colon segment using hematoxylin and eosin (H&E) and Masson trichrome (MT)

**I. The depth of destruction and affected layers in the colon wall**	
0	No affect (normal wall)
1	Erosions of mucosa
2	Ulceration of submucosal
3	Ulceration of the muscularis propria
4	Deep ulceration of the adventitia
**II. The extent of damage and lesions of the colon wall at each colon’s layer**	
0	No damage
1	Focal lesion
2	Localized lesion
3	Extensive lesion
**III.The score for epithelialization of the colon wall**	
0	Absent of epithelialization
1	Mild epithelialization
2	Moderate epithelialization
3	Marked epithelialization
**IV. The score of inflammation at different the histological layers (tunica muscularis, submucosa, and lamina propria) of the colon wall**	
0	Absent
1	Mild
	Hemorrhage, Edema, neovascularization, lymphatics dilation, connective tissue proliferation, mononuclear cells, and Polymorph nuclear neutrophils infiltration
2	Moderate
	Hemorrhage, Edema, neovascularization, lymphatics dilation, connective tissue proliferation, mononuclear cells, and Polymorph nuclear neutrophils infiltration
3	Severe
	Hemorrhage, Edema, neovascularization, lymphatics dilation, connective tissue proliferation, mononuclear cells, and Polymorph nuclear neutrophils infiltration
**V. The collagen deposition score in anastomotic line**	
0	No evidence
1	Occasional evidence
2	Light scattering
3	Abundant evidence
4	Confluent cells or fibers

#### Immunohistochemistry

Colon tissue samples that had been fixed in paraffin were cut into sections approximately 4 micrometers wide and put on slides of glass coated with saline. The sections were deparaffinized using xylol and dehydrated with different concentrations of ethanol. The antigens were extracted using autoclaving at 120 °C and pH 6 for 10 min. Endogenous peroxidase activity was inhibited with 3% H_2_O_2_ for an additional 10 min. The samples were then treated with primary antibodies against the cluster of differentiation 31 **(**CD31, 1:100 dilution, PECAM-1, Mob034, Diagnostic Bio-systems, USA) and the vascular endothelial growth factor (VEGF, 1:200 dilution, CME 365 AK Bk, Epitomics Co., USA). The samples were incubated at room temperature for 60 min and then washed four times with phosphate-buffered saline (PBS). The samples were incubated with anti-rabbit secondary antibodies at room temperature for 30 min. Visualization was done using a three-diaminobenzidine tetrahydrochloride liquid (Dako) technique at 25 °C for five minutes. Lastly, the sections of the sample were stained with hematoxylin [34].

### Statistical analyses

IBM SPSS Statistics 29 (Armonk, NY: IBM Corp) was used to analyze the data. The Kolmogorov-Smirnov test was used to detect the normality of numerical variables. The quantitative data were represented as mean ± and standard deviation, while the qualitative data were represented as numbers and percentages. The relationship between the two qualitative factors was analyzed using the chi-squared test. To compare continuous variables within the various variable measurements and between the groups, a repeated-measures ANOVA test was employed. Then, pairwise comparisons between statistically significant measurements were performed using the Bonferroni test. The minimum significant value for a statistically significant was set at *P* ≤ 0.05.

## Results

### Post-operative clinical evaluation

The general health and physical condition of the rabbits in both groups were stable, and none of the animals experienced a fever. The rabbits’ activities, urination, and defecation were all normal. There were no postoperative complications during the cutaneous wound healing at the surgical sites.

**Fig. 4:**
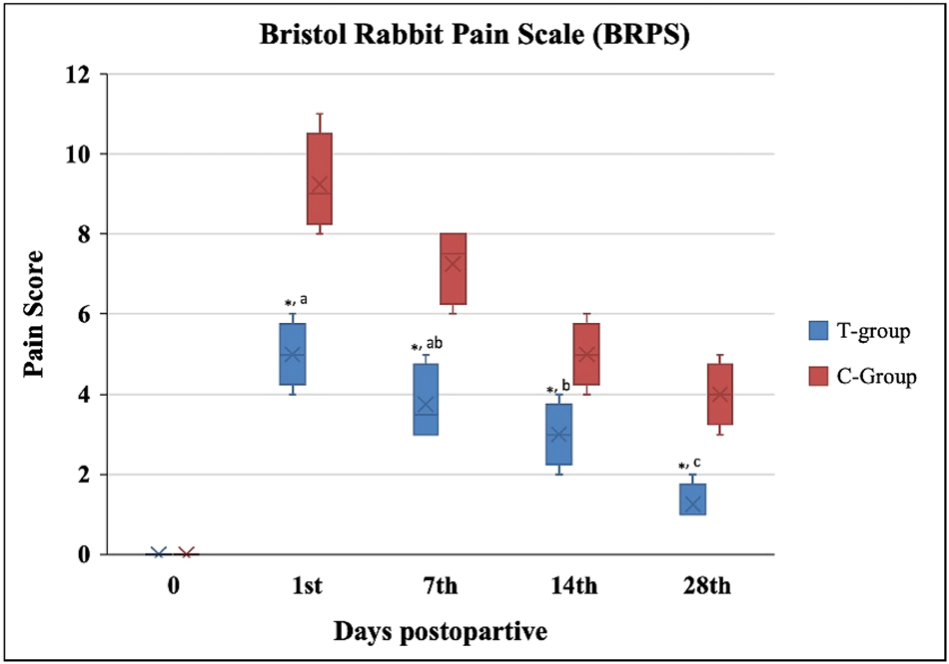
Bristol Rabbit Pain Scale (BRPS) between the control (C) groups and the AgNPs-CS/L-PRF treated (T) group the day before surgery (day 0) and on the 7th, 14th, and 28th days post-operations

Pain scores (BRPS) showed a sustainable decrease in the T-group (*p* ≤ 0.05) post-surgery than in the C-group (Fig. 4).

**Fig. 5:**
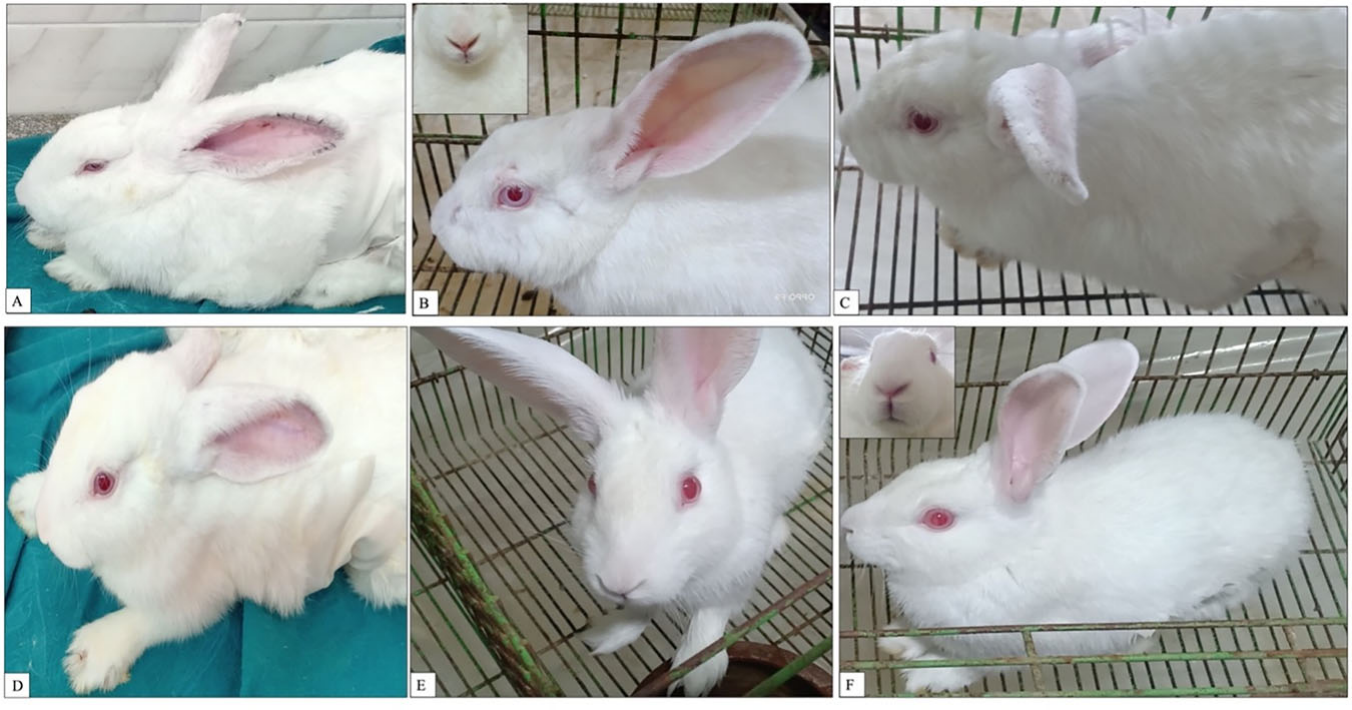
The Rabbit Facial expression according to the Bristol Rabbit Pain Scale (BRPS). **A–C** Rabbits in the C-group were dull, hunched backs, their Squinting, semi-closed eyes, nasal flare, and their ears directed backward. **D–F** Rabbits in the T-group were awake, lying with semi-closed eyes, and their ears slightly moved towards sounds

In both groups, the rabbits exhibited severe signs of pain 24 h post-operation, which were evident in their facial expressions. The signs gradually decreased in the following days post-operatively. The rabbits in the C-group were dull and showed no response to their surrounding environment. They had hunched backs, closed eyes, and ears that did not move when their heads were turned, scoring an eight (Fig. 5A–C). In contrast, the rabbits in the T-group were awake but showed little interest in their surroundings. They were lying with semi-closed eyes, and their ears slightly moved towards sounds, scoring a four (Fig. 5D–F).

### Post-mortem findings

**Fig. 6:**
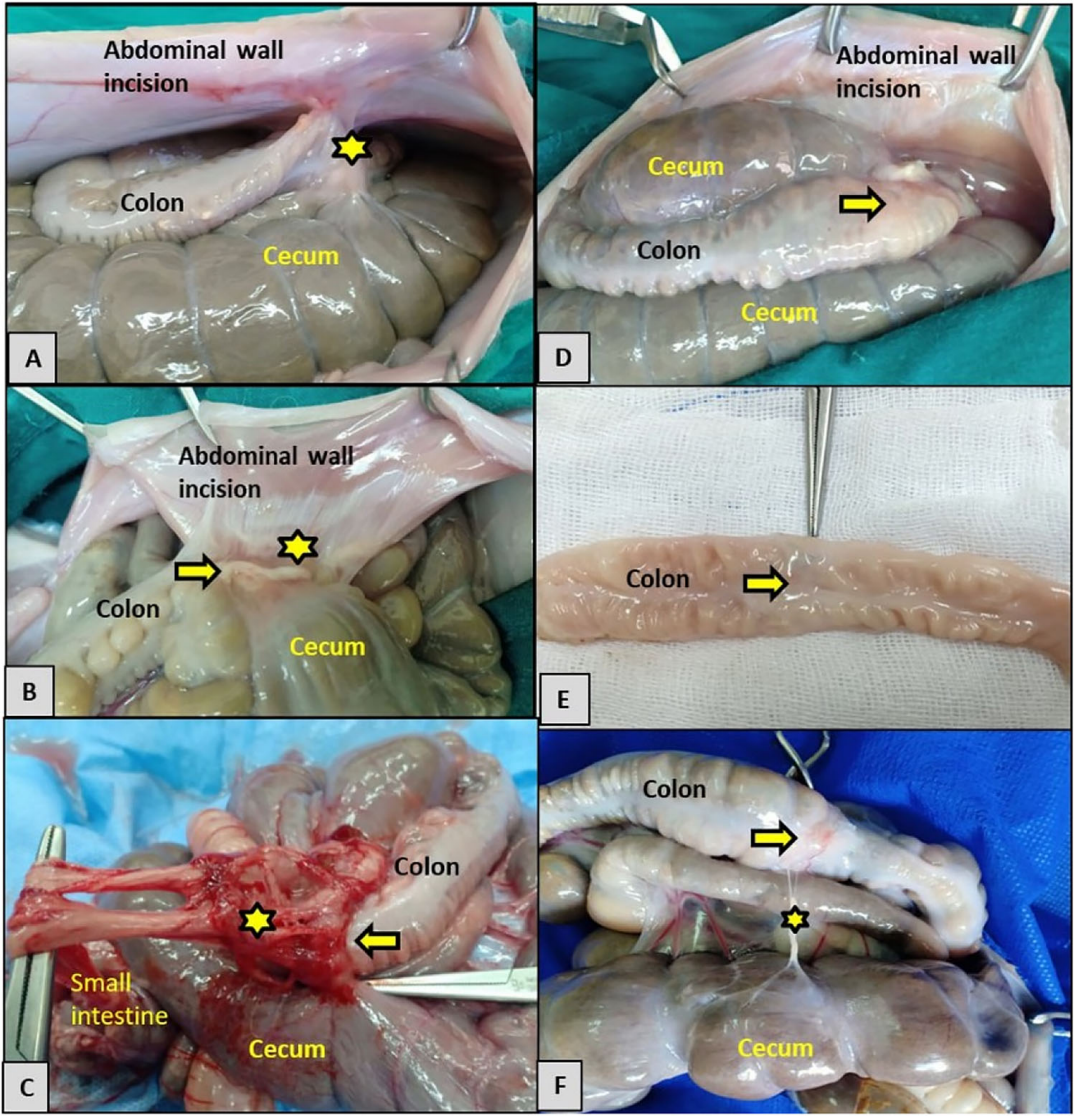
Adhesion grades in the control (C) group and the AgNPs-CS/L-PRF treated (T) group (D-F) groups. **A** Grade 2, soft, vascularized adhesion (stars) between colon anastomosis area (yellow arrow) and abdominal wall incision and cecum. **B** Grade 3, vascularized, solid (star) adhesion. **C** Grade 4, vascularized, solid, conglomerate adhesion (star) between the anastomotic line (yellow arrow), small intestine, and cecum. **D**, **E** Grade zero adhesion, **F** grade 1 easy, detached filamentous adhesion (star) between the anastomotic line (yellow arrow) and the cecum

The adhesion score significantly increased (*P* ≤ 0.002) in the C-group than in the T-group at different time points. In the C-group, five rabbits had an adhesion score of 2 (23.8%). Two adhesions were detected at the anastomotic incision between the colon and the abdominal wall. Seven rabbits (33.3%) had an adhesion score of three, indicating more than two adhesions between the anastomotic incision, the cecum, and the abdominal wall. The remaining nine rabbits (42.9%) had an adhesion score of 4, with the colon, small intestine, and cecum completely adhering to each other. In the T-group, fifteen rabbits (71.4%) had an adhesion score of zero, allowing the colon anastomotic wound to move freely without any adhesions. The remaining six rabbits (28.6%) had an adhesion score of one, in which one adhesion between the abdominal wall and the anastomotic colon (Fig. 6 and Table 2).

**Table 2:** The adhesion degree and numbers in control (C) and AgNPs-CS/L-PRF treated (T) groups

Adhesion Degree	0	1	2	3	4	Total	*P*-value
**C-group**	0 (0%)	0 (0%)	5 (23.8%)	7 (33.3%)	9 (42.9%)	21 (100%)	0.001
**T-group**	15 (71.4%)	6 (28.6%)	0 (0%)	0 (0%)	0 (0%)	21 (100%)	
**Total**	15	6	5	7	9	42	

Mean values and standard error. The letter (a and b). The comparison between the C and T-groups is shown by the *P*-value

### Anastomotic bursting pressure (BP)

The bursting pressure of the anastomotic segments showed a statistically higher (*P* < 0.001) in the T-group than the C-group at the 7th, 14th, and 28th days postoperatively. Additionally, there was a substantial increase (*P* < 0.01) in BP within the same group when compared to other time points. Additionally, post-hoc analysis employing the Bonferroni test was conducted to identify the differences between the two groups. The BP was considerably lower (*P* < 0.01) on the 7th day post-surgery than it was on the 14th and 28th (Table 3).

**Table 3:** The bursting pressure (BP) and Stenosis Degree (SD) in the control (C) group and AgNPs-CS/L-PRF treated group at different time points

	Groups	7th day	14th Day	28th day	*P*-value^1^	*P*-value^2^
**BP**	**C**	70.50 ± 34.92^a^	102.3 ± 22.51^a^	153.0 ± 22.49^a^	< 0.001	0.01
	**T**	175.2 ± 12.98^b^	231.7 ± 18.66^*,b^	299.3 ± 0.816^*,b^		
**SD**	**C**	69.33 ± 2.582^a^	61.33 ± 3.266^a^	52.67 ± 2.582^a^		0.03
	**T**	38.67 ± 2.160^b^	29.67 ± 3.204^*,b^	12.83 ± 1.722^*,b^		

Mean values and standard error. The small superscript letters (a and b) are significantly different between the C and T-groups at *P*-value^1^. The star (*) indicates that there is a significant difference from the 7-day time point at the *P*-value^2^

### Stenosis degrees (SD)

**Fig. 7:**
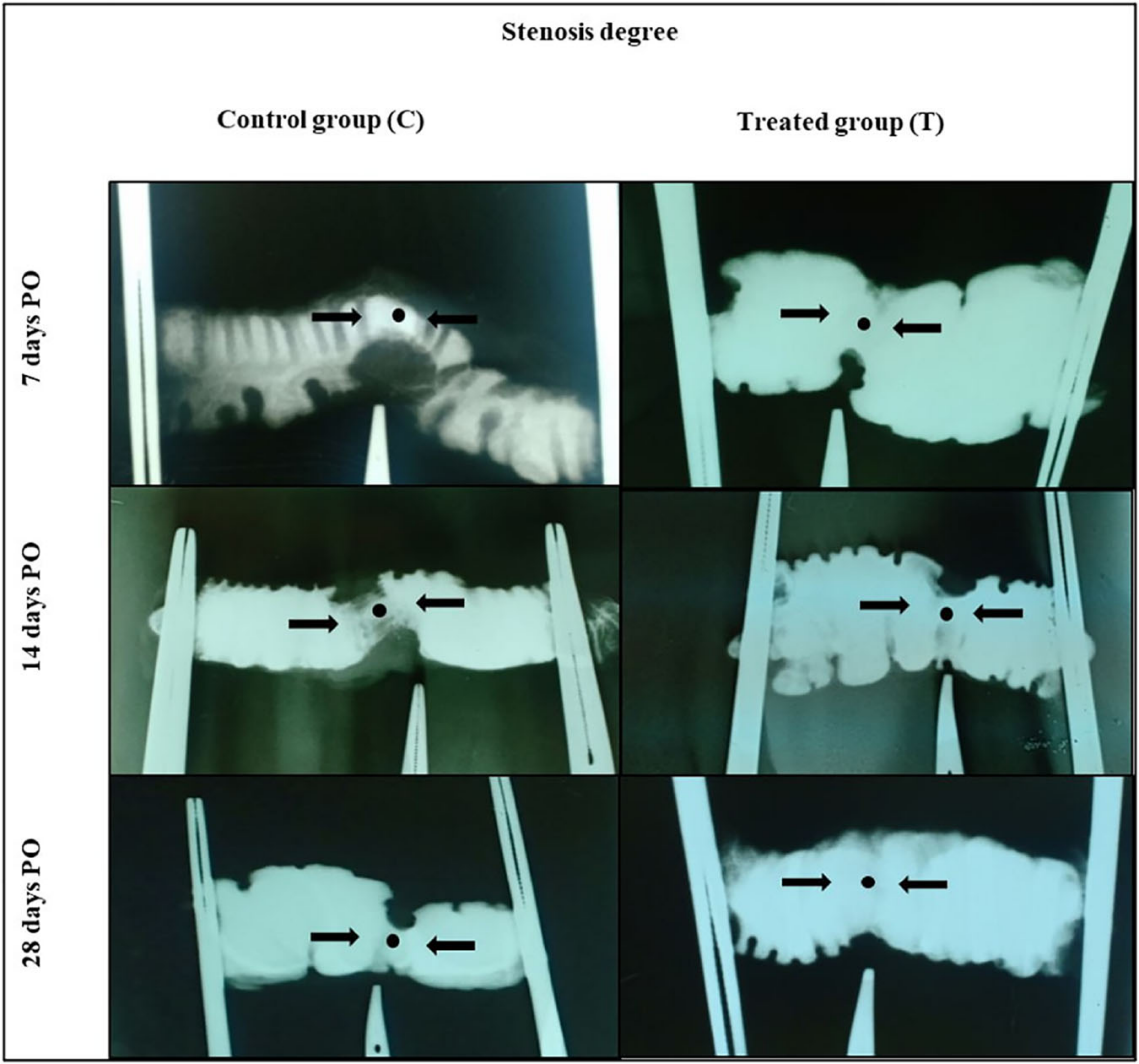
The degrees of stenosis of the anastomotic line (black dots) were evaluated post-contrast radiographic scanning in the control (C) group and the AgNPs-CS/L-L-PRF treated (T) group at the different time points postoperative

At different time points measured, the degree of stenosis in the T-group was significantly lower than that of the C-group. Furthermore, the degree of stenosis notably increased in both groups on the 7th day post-surgery, then on the 14th day post-surgery, and achieved the lowest values on the 28th day following surgery (Fig. 7 and Table 3).

### Histopathological findings

There was a substantial difference in the damage to the colon wall layers and the extensive defects between the groups (*P* < 0.011). The C-group had more severe damage with lymphatic dilation, edema, hemorrhage, mononuclear cells, and neutrophil infiltration. Additionally, extensive and widespread ulcers and lesions were observed in the anastomotic line of the colon wall in the adventitia (*n* = 6; 28.6%), muscularis (*n* = 7; 33.3%), submucosal layer (*n* = 5; 23.8%), and mucosa (*n* = 3; 14.3%) in the C-group. In contrast, the T-group showed a localized lesion in the mucosal (*n* = 3; 14.3%) and submucosal layers (*n* = 4; 19%) of the colon, while the remaining fourteen (n = 14; 66.7%) had no mucosal damage. The severity of edema, inflammatory cell infiltration, hyalinization, calcification, and necrosis in the anastomotic colon wall layers are detailed in (Table 4).

**Table 4:** Degrees of histological lesions in the anastomotic line of the colon layers between the control (C) and AgNPs-CS/L-PRF treated (T) groups at different time points

Histomorphological examination score						
Groups Postoperative days	C	T	C	T	C	T
	7th day		14th day		28th day	
Item	Lamina propria					
Mononuclear cells (MNC) infiltration	2	1	2	1	1	0
Polymorph nuclear cells (PMNC) infiltration	3	1	2	1	0	1
Edema	1	0	0	0	1	0
Hemorrhage	1	0	1	0	0	0
**Submucosa**						
PMNC infiltration	2	1	2	1	2	1
MNC infiltration	3	3	2	1	2	1
Edema	1	1	1	0	0	0
Hemorrhage	1	0	1	0	0	0
Propagation of connective tissues	1	2	1	2	2	3
Neoangiogenesis	1	2	2	2	2	2
Lymphatic dilatation	2	1	0	0	0	0
**Tunica muscularis**						
PMNC infiltration	2	1	2	2	3	2
Necrosis	2	1	3	2	2	2
Calcification	0	0	0	0	2	0
Hyalinization	2	1	2	0	0	0
Microbial aggregates	0	0	0	0	0	0
Hemorrhage	1	0	0	0	0	0

**Fig. 8:**
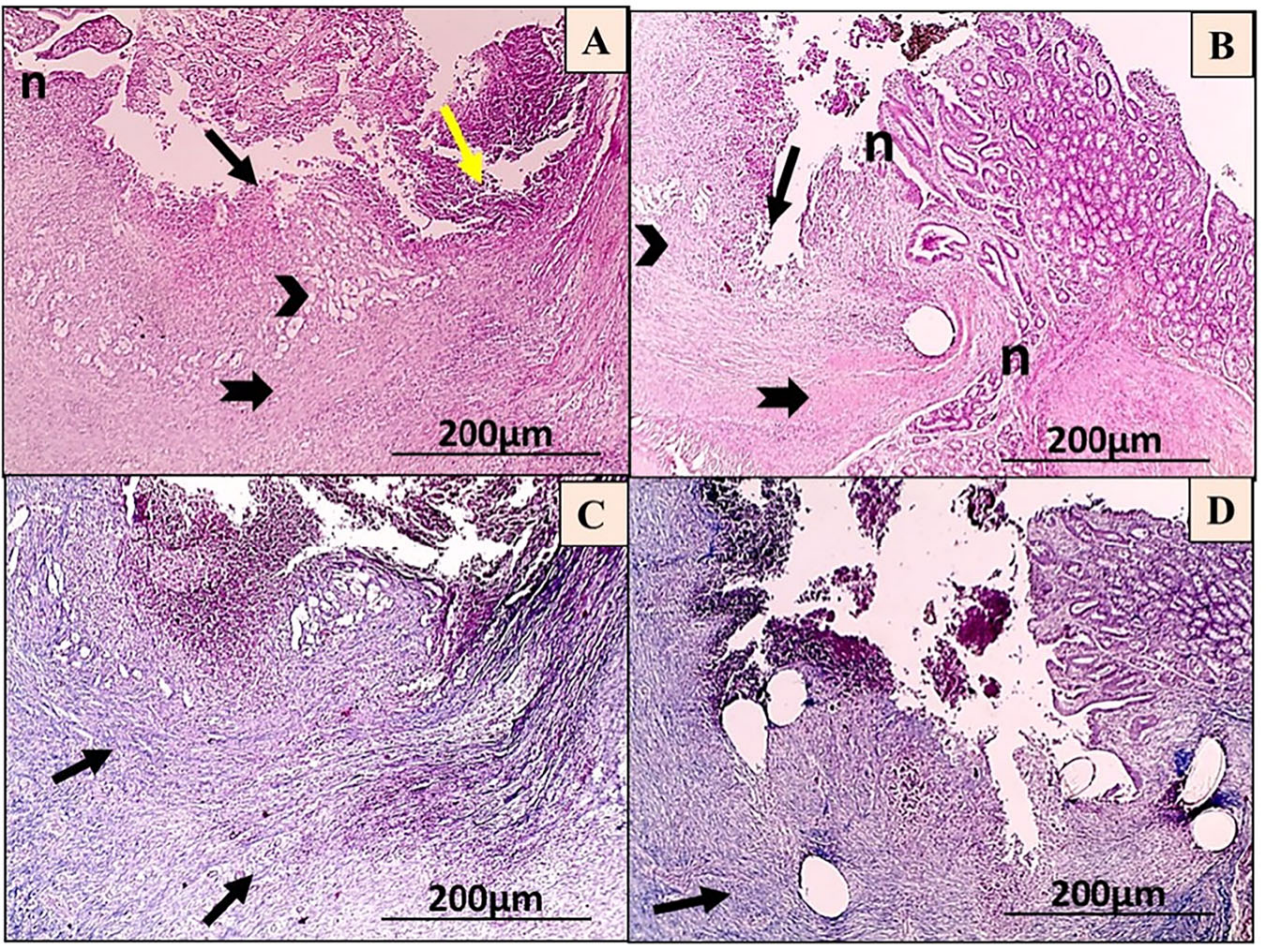
Microscopic images of anastomosis wounds in the control (C) group (**A**, **C**) and AgNPs-CS/L-PRF treated (T) group (**B**, **D**) at day 7 post-operation. The wound shows extensive polymorph nuclear cell infiltration (yellow arrows) throughout the layers of the colon, deep ulceration that reaches adventitia (black arrow), and minimal granulation tissue formation (black arrowhead) (**A**). Black arrowhead-shaped granulation tissue development with noticeable re-epithelialization (n) (**B**) (HE; X:40). As well, the wound displays scattered bluish stained fibrils of collagen (black arrow) (**C**), fine bluish stained collagen forming thin bundles (black arrow) (**D**) (MT; X:40)

On the seventh day post-operation, the H&E images showed that the C-group had a significant inflammatory response, minimal granulation tissue formation, and deep ulceration that reached the tunica muscularis. Unlike, the T-group showed the development of granulation tissue infiltrated with MNCs and noticeable re-epithelialization (Fig. 8A, B). Moreover, the MT images revealed that the C-group had dispersed bluish-stained collagen fibrils, while the T-group had narrow bundles of bluish-stained collagen deposition (Fig. 8C, D).

**Fig. 9:**
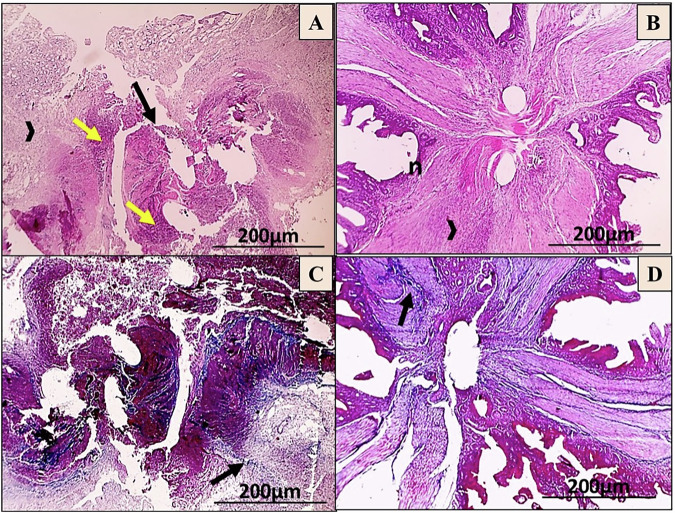
Microscopic images of anastomosis wounds in the control (C) group (**A**, **C**) and AgNPs-CS/L-PRF treatment group (**B**, **D**) at day 14 post-operation. The wound shows enhanced deposition of collagen (thick black arrow) in the submucosa, along with the formation of granulation tissue (black arrowhead), substantial infiltration of PMN (yellow arrow), and ulceration that reaches the Submucosa (black arrow) (**A**). Focal ulceration with increased re-epithelialization (n) that reaches the lamina propria (black arrow, thin one), greater collagen deposition (thick black arrow) is observed with granulation tissue formation (black arrowhead) (**B**) (HE; X:40). In addition, less bluish-stained collagen fibrils (black arrows) were found in the lamina propria and submucosa of the wounds (**C**), Higher and denser bluish bundles of collagen deposition (black arrow) appear in submucosa (**D**) (MT; X:40)

On the 14^th^ day post-surgery, the C-group revealed extensive ulceration in the submucosa layer along with the beginning of re-epithelialization, infiltration of PMN, formation of granulation tissue, and deposition of collagen. In contrast, the T-group showed that focal ulceration reached the lamina propria, with a moderate degree of re-epithelialization and deposition of collagen, as seen through H&E staining (Fig. 9A, B). MT staining revealed that the C-group had less bluish-stained fibril deposition of collagen in the lamina propria and submucosa, whereas the T-group exhibited an excess of bluish collagen with parallel bundles in the submucosa (Fig. 9C, D).

**Fig. 10:**
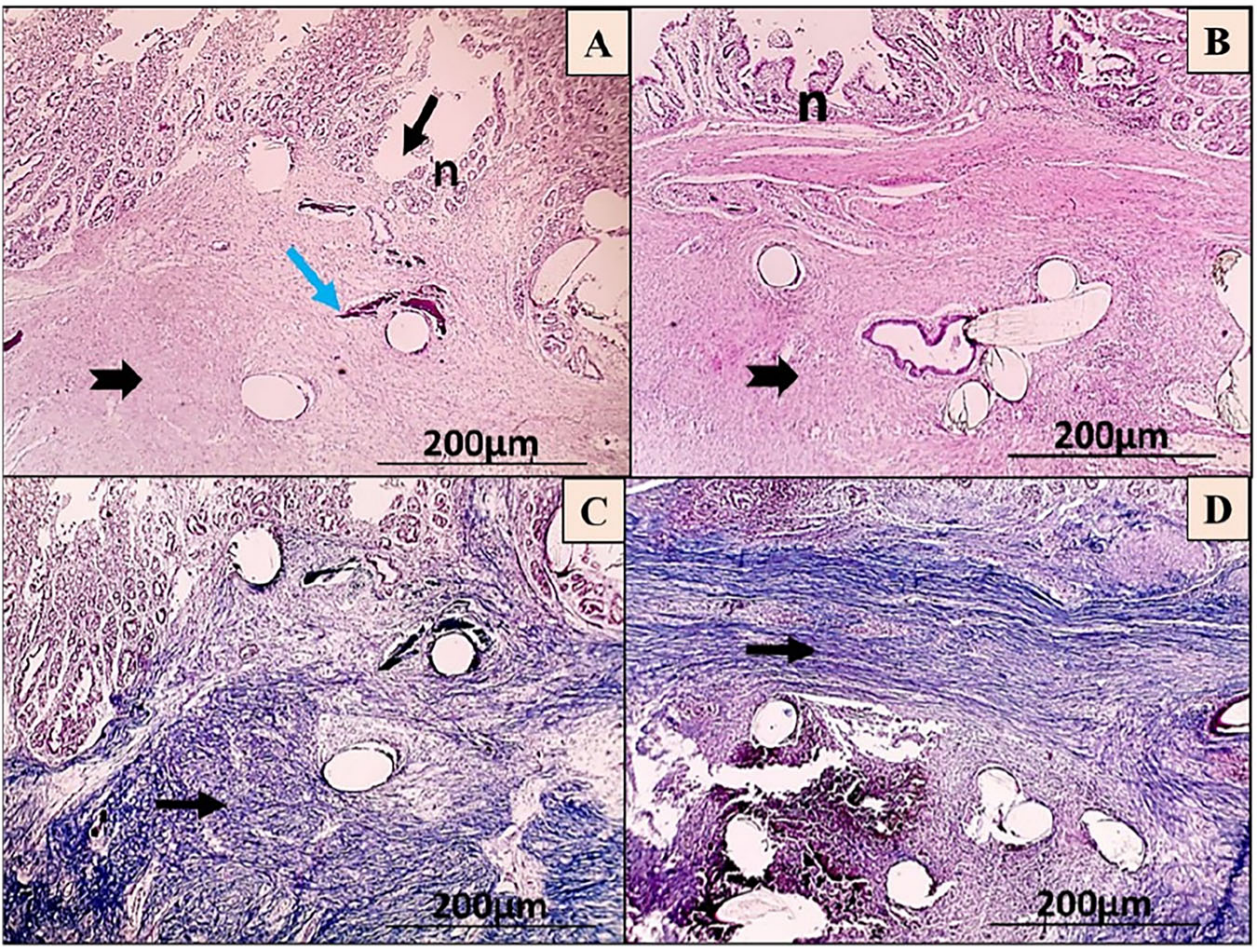
Microscopic images of anastomosis wounds in the control (C) group (**A**, **C**) and AgNPs-CS/L-PRF treatment (T) group (**B**, **D**) at day 28 post-operations. The wound exhibits focal calcification (blue arrow) in the submucosa and focal ulceration that reaches the lamina propria (thin black arrow), Prominent connective tissue deposition (thick black arrow) with increased re-epithelialization (n) (**A**). Re-epithelialization (n) is observed to be higher, with some MNCs penetrated by prominent connective tissue deposition (thick black arrow) (**B**) (HE; X:40). Also, the C-group had randomly ordered bluish collagen bundles (black arrow) (**C**), while the T-group had higher and denser bluish collagen bundle deposition (black arrow) in the submucosa (**D**) (MT; X:40)

On the 28^th^ day post-surgery, H&E staining exhibited that the C-group had an increase of re-epithelialization, connective tissue deposition, and focal ulceration that reached the lamina propria along with focal calcification in the submucosa. In contrast, lesions in the T-group showed stronger re-epithelialization and more prominent connective tissue deposition infiltrated with some MNCs (Fig. 10A, B). MT staining in the C-group displayed randomly ordered bluish collagen bundles, while the T-group had higher and denser bluish collagen bundle deposition in the submucosa (Fig. 10C, D).

The re-epithelialization and collagen deposition significantly differed (*P* < 0.001) between the C- and T-groups. These differences were significantly decreased in the C-group compared to the T-group at different time points measured (Table 5).

**Table 5:** Re-epithelialization and collagen deposition in the control (C) and AgNPs-CS/L-PRF treated (T) groups at different time points

Items	Group	7th day	14th day	28th day	*P*-value^1^	*P*-value^2^
**Neo-epithelialization**	**C**	0.00 ± 0.00^a^	0.50 ± 0.548^a^	1.17 ± 0.408^a^	<0.001	0.0473
	**T**	1.17 ± 0.408^b^	2.00 ± 0.632^*,b^	2.67 ± 0.516^*,b^		
**Collagen deposition**	**C**	1.00 ± 0.00^b^	1.83 ± 0.753^b^	2.33 ± 0.516^b^	0.001	0.038
	**T**	1.50 ± 0.548^b^	2.83 ± 0.753^b^	3.83 ± 0.408^b^		

Mean values and standard error. The small superscript letters (a and b) are significantly different between the C and T-groups at *P*-value^1^. The star (*) indicates that there is a significant difference from the 7-day time point at the *P*-value^2^

### Immunohistochemistry

**Fig. 11:**
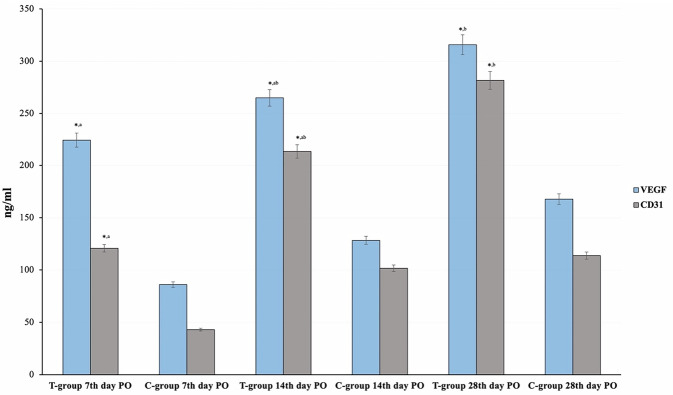
The Vascular endothelial growth factor and the cluster of differentiation 31 (ng/ml) between the control (C) group and AgNPs-CS/L-PRF treatment (T) group at the 7th, 14th, and 28th days postoperative The smallsuperscript letters are significantly different between the C and T groups. The star (*) indicates that there is a significant difference from the 7-day time point at *P* 0.01

**Fig. 12:**
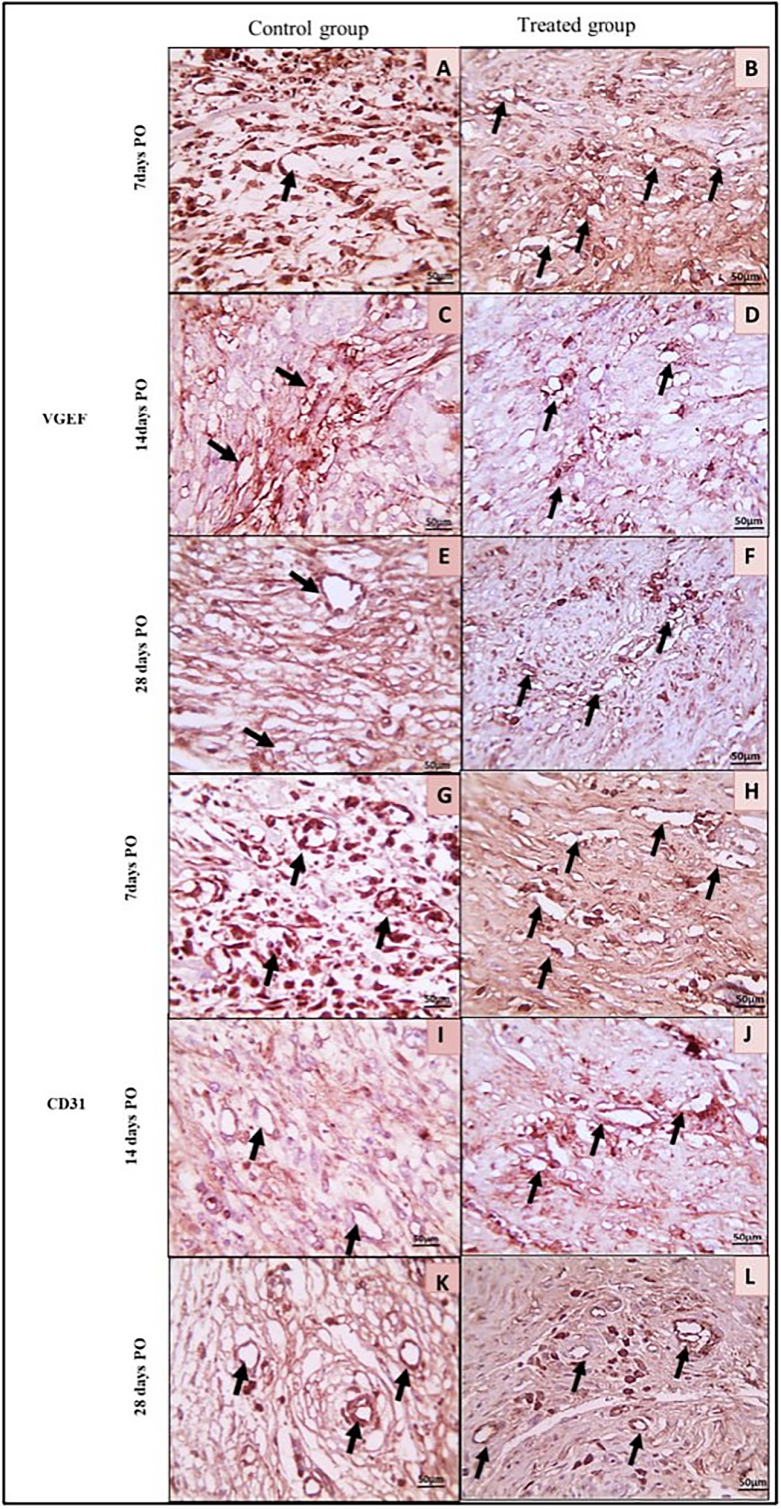
Immunohistochemical views of colon anastomotic wounds in the control (C) group and AgNPs-CS/L-PRF treated (T) group in rabbits at 7-, 14-, and 28-days post-operative against VEGF (**A**–**F**) and CD31 (**G**–**K**). Control wounds (**A**, **C**, **G** & **I**) showed mild positive brown immunostaining while (**E** & **K**) revealed a moderate positive brown immunostaining for a few dilated capillaries (black arrows). Treated wounds (B and H) showed a moderate positive brown immunostaining, while (**D**, **F**, **J**, and **L**) showed a considerable number of tiny capillaries with a strong positive brown immunostaining (black arrows). (IHC counterstained with Mayer’s hematoxylin. X: 400)

On day 7^th^, the VEGF and CD31 antibodies expression scores were significantly lower (*P* < 0.001) in the C-group than in the T-group (86.33 ± 12.11 and 43.00 ± 10.48 versus 224.2 ± 16.25 and 120.8 ± 4.92 ng/ml respectively). Additionally, on the 14th postoperatively, the VEGF and CD31 antibodies expression scores significantly (*P* < 0.001) declined in the C-group compared to the T-group (128.3 ± 11.69 and 101.7 ± 10.33 versus 265.0 ± 18.71 and 213.3 ± 12.11 ng/ml respectively). Likewise, on the 28th postoperatively, the VEGF and CD31 antibodies expression scores were significantly reduced (*P* < 0.001) in the C- than the T-group (167.7 ± 10.33 and 113.8 ± 10.21 versus 315.8 ± 9.1 and 231.7 ± 19.41 ng/ml respectively) (Fig. 11). Both groups showed positive brown immunostaining for VEGF and CD31 antibodies (Fig. 12).

## Discussion

Anastomotic leakage and poor healing of the anastomotic line are among the most serious complications in gastrointestinal (GI) tract surgery, particularly in colon anastomoses [3]. There have been reports of colon anastomoses leaking up to 30% of the time [4] making it the surgical model used in the present study. In the past decade, there has been a tendency to use various agents with anti-inflammatory properties, like L-PRF [35] and AgNPs [36], as they have been considered viable methods to enhance tissue repair and regeneration. Preventing leakage from anastomotic wounds is challenging and may require multiple laparotomies. The results of previous treatments during wound healing were insufficient, and the rates of morbidity and mortality are still very high [37]. Preventing anastomotic leakage is more crucial than treating it [8]. Consequently, the main goal of our study was to apply new treatment materials that improve the healing of colon anastomotic wounds. The anastomotic colon was wrapped L-PRF loaded with AgNPs in an attempt to reduce or prevent various complications such as leakage, adhesion, stricture development, and fistulas. Furthermore, our study aimed to reduce the severity of adhesions, enhance tensile strength, promote angiogenesis, and boost collagen deposition in the transected colon.

Rabbits were chosen as animal models in this study because they are large enough to be easily handled in laboratory settings, allowing for various types of procedures and interventions. Additionally, their size makes them suitable for surgical experiments [38]. Moreover, rabbits have physiological and anatomical features that are similar to those of humans, making them valuable for studying human diseases and treatments [39].

The L-PRF is superiorly used for many reasons, such as its ability to keep growth factors active, protect them from proteolysis for a longer time, and effectively promote tissue regeneration [40]. The advantages of using autologous L-PRF for reinforcing colon anastomotic wounds were mentioned [41–43]. These advantages were simple preparation, cost-effectiveness, and minimal skill requirements. Additionally, autologous L-PRF contains a dense fibrin biomaterial that can protect growth factors (GFs) from proteolysis, allowing for the slow release of large amounts of growth factors. This contributes to tissue regeneration and healing in the anastomotic areas. Moreover, autologous L-PRF was used in clot form to cover the anastomosis site, which can prevent the chance of leakage and act as a barrier material. Furthermore, there are rare immunologic rejections and complications.

Furthermore, it provides a special thick fibrin biomaterial that efficiently promotes tissue regeneration and allows for the progressive release of several growth factors [44]. AgNPs are the most well-known nano product [18] and have antimicrobial effects with long-term anti-inflammatory properties [19]. However, they require other loaded media to prevent the overgrowth of the silver particles. Thus, in this study, CS is chosen as a loaded material. Additionally, CS is a biopolymer with superior biodegradability, antibacterial activity, analgesic qualities, and rapid wound-healing capabilities. This strongly suggests using it as a novel biomatrix for clinical purposes [45, 46]. Furthermore, previous research has shown that there are synergistic effects between certain biological preparations and nanoparticles such as L-PRF, and AgNPs respectively [45, 46].

Adhesions are considered one of the most challenging complications that can result from colon surgery and they are also a main cause of intestinal obstruction and chronic abdominal discomfort [47]. Consequently, the primary goal of many previous strategies has been to reduce the occurrence and severity of adhesions, while also maintaining the natural healing process and preventing infection [48].

Our results indicated that treatment with augmented AgNPs loaded on CS and L-PRF scaffold significantly reduced the adhesion score degree around the anastomosis compared to the C-group. This effect is crucial during the normal healing process, as adhesions can lead to poor functional outcomes [49]. Inhibition of inflammation plays a vital role in preventing adhesion [50]. The anti-inflammatory characteristics of L-PRF may be attributed to the presence of TGF-β1and PDGF [51]. Furthermore, CS has an essential role in regulating both pro- and anti-inflammatory cytokines [52]. Therefore, the anti-inflammatory effects of AgNPs and L-PRF [19] may aid prevent the formation of adhesions in the T-group.

The radiographic examination images revealed that both groups had stenosis, with the highest luminal stenosis scores in the C-group (69.33 ± 2.582) on the 7th day PO and the lowest level of luminal stenosis scores in the T-group (12.83 ± 1.722) on the 28th day PO. The large adhesion that caused compression and prevented the lumen from expanding at the suture line may have been the cause of the increased percentage of lumen stenosis in the C-group. On the other side, minimal stenosis in the T-group can result from minimal adhesion formation; this result was supported by who stated that the risk of adhesion and subsequent bowel stenosis and obstruction may be reduced by covering the anastomosis site with barrier materials. Therefore, the low amount of adhesion is not only due to the anti-inflammatory effects of AgNPs and L-PRF but also the greater hemostatic effect of L-PRF. Furthermore, one of the features of CS is its hemostatic potential to prevent postoperative intra-abdominal bleeding, which is a stimulus for adhesion [53].

The BP is considered a more accurate measurement that indicates the physiological strength of intestinal tissue. The T-group had better mechanical strength, with the anastomosis BP increasing almost twofold. This may be attributed to several reasons. First; a higher collagen deposition during tissue healing after anastomosis [54] as appeared in histological examination. Second, the AgNPs-CS/L-PRF combination has an essential role in inhibiting or preventing local complications, while also supporting and promoting the healing process of the anastomotic colon wound. The ability of both AgNPs-CS/L-PRF to prevent local complications may be attributed to the rapid formation of a fibrin network by L-PRF, which encourages hemostasis [55]. Third, the superior ability of L-PRF to reduce signs of infection and inflammatory cascades is due to the varying amounts of leukocytes in L-PRF that have a bactericidal impact [56]. Fourth, AgNPs have excellent broad-spectrum antimicrobial properties as mentioned [57], suggesting that metal nanoparticles could be used as effective bactericidal materials.

On histopathological examination, the group treated with a combination of AgNPs-CS/L-PRF had better histologic scores showing increased formation of new vessels, a higher amount of collagen deposition, and earlier re-epithelialization compared to the C-group. These results may be attributed to the excellent biocompatibility, suitable biodegradability, and promising antimicrobial activities of both materials. Therefore, they act as a shield against adhesions, diminish inflammation, and promote the healing process [12, 19].

L-PRF and AgNPs-CS significantly enhance the quality of wound healing and can work synergistically to promote proper homeostasis and reduce inflammation. The beneficial therapeutic effects of L-PRF are primarily attributed to the wide range of platelet-derived protein molecules. These molecules include growth factors, cytokines, and other bioactive peptides that can be released to stimulate cell migration and proliferation, fibrin matrix remodeling, and the secretion of a cicatricial collagen matrix [58], consequently hastening the wound healing process [59]. Additionally, AgNPs have proven to be more efficient in preventing the infiltration of inflammatory cells, suppressing these cells, and down-regulating pro-inflammatory cytokines. This has resulted in hastened tissue repair, improved remodeling, and wound healing [54].

Immunohistochemical staining and capillary vascular count for angiogenic factors VEGF and CD31, as well as proper morphological analysis, revealed notably higher vascular counts and angiographic scores in the T-group. This may be attributed to the higher amount of GFs provided to the wounds over a prolonged period, while these rates were limited in the C wounds [60]. This explanation revealed the essential role of L-PRF in providing a strong angiogenic factor that is important for the healing process [60]. The nanoscale form of Ag helped maintain GFs at the anastomotic wound for a longer period. The biodegradable AgNPs-CS allow for the gradual release of these GFs and other healing stimulators at the target site over an extended period, ranging from days to weeks [19]. However, complete resorption of the L-PRF clot was not achieved after 14 days PO.

In addition, CS also has a pro-angiogenic effect on wounds, leading to higher levels of microvascular density in wound tissues. CS can stimulate the expression of VEGF in wound tissues, subsequently increasing the development of blood vessels in wounds [61]. Therefore, the T-group demonstrates a stronger and faster rate of colon anastomosis repair due to enhanced angiogenesis [59].

In contrast to previous studies [54] that mentioned the potential toxicity of any nanomaterial, this study did not record any cytotoxic effects of AgNPs. This could be explained by the minimal quantity of silver used which was diluted by body fluids (L-PRF) [19].

There were two main limitations of this study. Firstly, differences exist between animal models and the human body. Further exploration is needed to understand the effectiveness and stability of the scaffold in the human digestive tract environment. This includes factors like intestinal flora, physiological peristalsis, and tissue microenvironment. Furthermore, the preparation process and application method should be optimized according to the actual situation to be applicable to clinical practice. Secondly, The safety of long-term residue of nanomaterials in vivo and the potential related toxicity, especially with the long-term survival of tumor patients after radical surgery. Therefore, further relevant experimental studies on chronic toxicity and cumulative organ effects should be done to consider the safety of future clinical applications.

## Conclusions

Wrapping the colon anastomotic wounds with L-PRF augmented with AgNPs-CS promotes the healing processes of the colon by strengthening the anastomosis and preventing postoperative complications. L-PRF is a completely biodegradable, safe, easy to prepare and apply, and non-toxic. AgNPs-CS displayed a set of unique biological properties and also revealed synergistic effects with L-PRF. Therefore, AgNPs-CS/L-PRF could protect high-risk patients from anastomotic leaks following bowel procedures. The combination of AgNPs loaded into a CS solution and inoculated in an L-PRF scaffold could be considered a beneficial biological scaffold material for colon anastomotic wounds in experimental rabbits.

## Data Availability

The authors confirmed that the data supporting the results of the current study, and its supplementary materials are available from the corresponding author on reasonable request.

## Abbreviations

**L-PRF**: Leukocyte-Platelet-Rich Fibrin.
**CS**: Chitosan
**CS/AgNPs**: Chitosan silver nanoparticles
**TEM**: Transmission electron microscope
**UV**: Ultraviolet
**ICP**: Inductively coupled plasma
**BP**: Bursting pressure.
**SEM**: Scanning Electron Microscopy analysis.
**SD**: Stenosis Degree.
**VEGF**: Vascular endothelial growth factor
**CD 31**: Cluster of differentiation
**PMNs**: Polymorph nuclear neutrophils.
**CCD**: Charge-coupled device
**SAED**: selected area electron diffraction
